# Critically ill patients with infective endocarditis, neurological complications and indication for cardiac surgery: a multicenter propensity-adjusted study

**DOI:** 10.1186/s13613-023-01221-x

**Published:** 2024-02-02

**Authors:** Alexandre Gros, Benjamin Seguy, Guillaume Bonnet, Yves-Olivier Guettard, Xavier Pillois, Renaud Prevel, Arthur Orieux, Julien Ternacle, Sebastien Préau, Yoan Lavie-Badie, Elisabeth Coupez, Rémi Coudroy, Delphine Marest, Raphaël P. Martins, Didier Gruson, Thomas Tourdias, Alexandre Boyer, the ICE-COCA investigators, Renaud Prevel, Pierre Coste, Hikaru Fukutomi, Bertrand Souweine, Sébastien Preau, Saad Nseir, Aurélia Toussaint, Olivier Outteryck, Jean Reignier, René Robert, Raphaël Martins, Jean Marie Urien, Lydie Porte, Guillaume Robin, Gaëtan Charbonnier, Benjamine Sarton, Stein Silva

**Affiliations:** 1 Service de Médecine Intensive Réanimation, CHU de Bordeaux, 33000 Bordeaux, France; 2 Service de Neuroradiologie, CHU de Bordeaux, 33000 Bordeaux, France; 3 Soins Intensifs de Cardiologie, Hôpital Cardiologique du Haut-Lévêque, CHU de Bordeaux, 33000 Pessac cedex, France; 4 Hôpital Cardiologique du Haut-Lévêque, LIRYC Institute, 33000 Bordeaux, France; 5 Service de Médecine Intensive Réanimation, Inserm, Institut Pasteur de Lille, U1167, University of Lille, CHU Lille, 59000 Lille, France; 6 Fédération de Cardiologie, Centre Expert de la Valve, CHU de Toulouse, 31000 Toulouse, France; 7 Réanimation Médicale Polyvalente, CHU de Clermont-Ferrand, 63000 Clermont-Ferrand, France; 8 Médecine Intensive Réanimation, CHU de Poitiers, F-86000 Poitiers, France; 9 Groupe ALIVE, INSERM CIC 1402, Université de Poitiers, F-86000 Poitiers, France; 10 Service d’Anesthésie-Réanimation, Hôpital Laënnec, CHU de Nantes, 44000 Nantes, France; 11 Cardiologie et Maladies Vasculaires, CHU de Rennes, 35000 Rennes, France; 12 INSERM-U1215, Neurocentre Magendie, 33000 Bordeaux, France

**Keywords:** Endocarditis, Ischemic stroke, Hemorrhagic stroke, Cardiac surgery, Intensive care

## Abstract

**Background:**

The benefit–risk balance and optimal timing of surgery for severe infective endocarditis (IE) with ischemic or hemorrhagic strokes is unknown. The study aim was to compare the neurological outcome between patients receiving surgery or not.

**Methods:**

In a prospective register-based multicenter ICU study, patients were included if they met the following criteria: (i) left-sided IE with an indication for heart surgery; (ii) with cerebral complications documented by cerebral imaging before cardiac surgery; (iii) with Sequential Organ Failure Assessment score ≥ 3. Exclusion criteria were isolated right-sided IE, in-hospital acquired IE and patients with cerebral complications only after cardiac surgery. In the primary analysis, the prognostic value of surgery in term of disability at 6 month was assessed by using a propensity score-adjusted logistic regression.

**Results:**

192 patients were included including ischemic stroke (74.5%) and hemorrhagic lesion (15.6%): 67 (35%) had medical treatment and 125 (65%) cardiac surgery. In the propensity score-adjusted logistic regression, a favorable 6-month neurological outcome was associated with surgery (odds ratio 13.8 (95% CI 6.2–33.7). The 1-year mortality was strongly reduced with surgery in the fixed-effect propensity-adjusted Cox model (hazard ratio 0.18; 95% CI 0.11–0.27; *p* < 0.001). These effects remained whether the patients received delayed surgery (*n* = 62/125) or not and whether they were deeply comatose (Glasgow Coma Scale ≤ 10) or not.

**Conclusions:**

In critically ill IE patients with an indication for surgery and previous cerebral events, a better propensity-adjusted neurological outcome was associated with surgery compared with medical treatment.

**Supplementary Information:**

The online version contains supplementary material available at 10.1186/s13613-023-01221-x.

## Background

The annual incidence of infective endocarditis (IE) is around 3.1 cases/100,000 inhabitants in France [1] and 7.4 cases/100,000 in the USA [2]. Many complications may occur during the clinical course of IE [3, 4], some of them requiring the patient to be admitted to intensive care unit (ICU). These include cardiac failure due to valvular lesions or prosthetic valve dysfunction, septic shock, and severe neurologic events. Surgery has consensual indications [5] and is needed in half cases of complicated IE [6, 7] which is associated with a reduction of mortality when performed early (emergency < 24 h or urgent < 7 days) in the IE course [8]. Presence of neurologic events is one of the most challenging conditions in the care of IE patients. Symptomatic neurologic events are frequent (52–69%) in critically IE patients [9–12], and are associated with a 45% mortality rate [13]. Neurologic events mainly include stroke (69–73%) and intracranial hemorrhage (27–49%) [10, 12]. The risk of hemorrhagic transformation of an ischemic stroke or worsening of an intracranial hemorrhage might be increased by the anticoagulation during cardiopulmonary bypass. Intraoperative hypotension may also worsen a pre-existing cerebral ischemia. The benefit–risk balance of surgery for IE with neurological failure remains debated.

The former attitude was to postpone surgery for 15 days after a neurological event [5]. However, a recent study suggests that an earlier surgery would be beneficial regarding the risk of subsequent complications, particularly the embolic events [8]. The guidelines of the European Society of Cardiology (ESC) indicate that cardiac surgery may be performed safely after ischemic stroke, provided that the patient has no extensive neurologic damage and no cerebral bleeding [5]. In case of intracranial hemorrhage, they recommend surgery to be postponed for at least 1 month, but “if urgent cardiac surgery is needed, close cooperation with the neurosurgical team is mandatory” [5]. However, studies on which these recommendations are based do not include many critically ill patients. In a prospective multicenter study including ICU patients, good functional outcomes (*n* = 24/30) were observed in patients with a pre-existing neurologic complication who underwent cardiac surgery even if the sample size was limited [10]. It was also demonstrated that patients with denied surgery despite indications have poor outcomes [11, 14]. If randomized studies are precluded for ethical concerns [15, 16], prospective observational studies with appropriate methods to limit confusion bias have not been conducted before and could add to the level of evidence.

We hypothesized that critically ill IE patients with an indication for cardiac surgery but presenting with cerebral complications may be the object of ethical limitations although they should still benefit from surgery. The main objective of this study was to compare the neurological outcome of these patients whether they received surgery or not.

## Methods

### Design

The ICE-COCA study (InfeCtious Endocarditis with Cerebral cOmplications: a Cohort of French reAnimations) is a multicenter study that included critically ill patients with acute IE admitted to surgical, cardiologic or medical ICU in seven French tertiary referral University Hospitals (Bordeaux, Lille, Toulouse, Nantes, Poitiers, Rennes and Clermont-Ferrand). Every patients with IE who was admitted to these centers was consecutively screened and included in a prospective registry in each center between January 2010 and July 2017.

### Inclusion/exclusion criteria

The inclusion criteria for this study were the addition of:

(i) Definite and active (admission within the first 30 days after the initiation of antibiotics) left IE according to the modified Duke criteria [17] with an indication for surgery.
(ii) Cerebral complications (symptomatic or not) documented by cerebral imaging before cardiac surgery.
(iii) Severity criteria defined as Sequential Organ Failure Assessment score (SOFA) ≥ 3 (to prevent including patients admitted to ICU more because of a deterioration risk than for an already existing severity), one of these justifying admission to ICU.

Exclusion criteria were isolated right endocarditis, in-hospital acquired endocarditis and patients who developed cerebral complications only after cardiac surgery.

### Data collection at inclusion

For every patient, the following data were collected: age, gender, history of endocarditis or valve surgery, and comorbidities (intravenous drug use, immunosuppression, diabetes mellitus and chronic kidney disease defined as glomerular filtration rate < 60 mL/min/1.73 m^2^). Care-associated IE was defined as early prosthetic valve IE (< 12 months post-surgery) or non-nosocomial healthcare-associated IE [5]. Acute patient’s condition was assessed by SOFA score and GCS score. The specie isolated from blood culture and/or heart valve from surgery culture was dichotomized as *Staphylococcus aureus* or other species according to previous prognostic studies [6]. Echocardiographic findings included the valve involved, the presence of vegetation and its maximum size and the presence of a severe regurgitation. Regarding therapy, the type of antibiotic was collected and the first day of antibiotic therapy was considered as IE day 0. Patients all met one of the three main indications for surgery (heart failure, uncontrolled infection, prevention of embolism) according to the 2015 guidelines [5]. The timing of surgery was quoted as either in line or delayed compared to the current recommendations according to the indications (emergency, urgent or elective) [5].

### Cerebral complications

Every patient was explored with brain imaging before surgery to assess cerebral complications. Cerebral computed tomography (CT)-scans or magnetic resonance imageries (MRIs) could be performed at the discretion of the investigating center according to availability and to the patient’s clinical condition. When multiple CT-scans were available, the one performed close to the date of surgery or the one that showed the largest lesion load was retained for analysis. When both CT and MRI were available, MRI was retained for analysis as long as the protocol included at least diffusion weighted imaging (DWI), T2*, fluid attenuated inversion recovery (FLAIR), and 3D-T1-weighted sequences acquisition. Injection of contrast agent (iodine or gadolinium-based) was not mandatory and was listed. All the scans were reviewed centrally at the coordinating center (Bordeaux University hospital) by a trained reader with 5 years of radiological experience, blinded from baseline and follow-up clinical data. This reported ischemic lesions, intraparenchymal hemorrhage, subarachnoid hemorrhage (SAH), brain abscess, and infectious intracranial aneurysm (IIA). Leukoaraiosis was quoted considering that this is a significant marker of small vessel disease whose severity (brain frailty) has been associated with functional outcome after stroke [18]. Microbleeds were also counted when MRI was available. Brain imaging has been described in a previous publication [19].

### Follow-up assessment

Respectively, at 6-month and one-year follow-up, neurological outcome (evaluated by quoting the modified Rankin score (mRs)) and mortality were recorded face-to-face or through a telephone interview with the patient or one of the family members, as validated [20].

### Statistical analysis

The primary outcome was neurological outcome at 6 months defined as good (mRS < 3 meaning the ability to walk without assistance) or poor otherwise (mRS ≥ 3) [21]. The primary analysis consisted in a propensity score-adjusted logistic regression assessing whether receiving surgery or not is an independent variable associated with the primary outcome (i.e., mRS at 6 months). A propensity score was used to adjust for possible patient selection bias attributable to nonrandomized assignment of surgery. As a matter of fact, ethical limitations at the origin of surgery contraindication are complex and include comorbidities, severity of cerebral complication, severity of shock if any, and the indication for surgery. Therefore, the most clinically and statistically relevant associations were selected for the final propensity model and included age, gender, valve prosthesis, aortic *vs* mitral *vs* both IE, SOFA score, septic shock, the nature of the cerebral complication (stroke vs hemorrhagic assuming that ischemic stroke complicated by hemorrhagic transformations of ECASS class 4 could be gathered with hemorrhagic stroke), and indication for surgery.

The 1-year mortality rate was analyzed as a secondary outcome. A selection bias—survivor treatment survival bias—is attributable to nonrandomized assignment of surgery and frequently occurs in this kind of studies. As the standard propensity analysis cannot fully address this bias, a time-dependent Cox regression analysis is useful. We thus performed a secondary analysis in which the propensity score estimates were used as a covariate in a Cox model to adjust the analysis of the association between 1-year mortality rates and surgery. We completed a 1:1 matching based on the nearest-neighbor matching algorithm with a caliper width of 0.2 of the propensity score with all nine variables in a secondary analysis. No difference was shown with a broader caliper of 0.4. A log rank test was computed to assess the association between surgery and mRankin score in this condition. The same approach was used for 1-year mortality. Finally, a multivariate survival logistic regression using an inverse probability of treatment weight (IPTW) estimator was run. These models have been extensively described elsewhere. Briefly, four steps were performed: first, a univariate analysis of baseline variables associated with surgery was performed to identify variables associated with *p* < 0.20. Second, these variables were introduced in a non-parsimonious multivariable logistic regression model to compute the inverse probability of treatment weights (IPTW) for individual patients. The steps one and two were performed to assess the probability of having a surgery. Third, weights were truncated at the 1st and the 99th percentiles to avoid an over-dispersion. Fourth, a multivariable logistic regression, using the IPTW and including variables which were pertinent or associated with the outcome, was performed to assess the risk of surgery on the primary outcome. Continuous variables were expressed as mean ± standard deviation (SD) or median (IQR) as needed. Categorical variables were expressed as proportion (%). Shapiro–Wilk test was used to test for normality. For continuous variables, independent-sample parametric (unpaired Student’s t-test) or non-parametric tests (Mann–Whitney) were used as appropriate. For categorical variables, Fisher’s exact or *χ*^2^ tests were used as appropriate. All statistical tests were 2-sided and a *p* < 0.05 was considered statistically significant. Statistical analyses were performed using IBM SPSS Statistics v17.0 software.

### Ethics

The study was approved by the ethics committee of *Société de Réanimation de Langue Française* (CE-SRLF 15–54). Furthermore, the study complies with the protection of personal health data and of private life within the framework provided for by the European Union General Data Protection Regulation. Data were anonymized and the database was approved by the national data protection authorities (declaration number 2082557 v0).

## Results

### Patients and IE characteristics

Two hundred and fifty-five patients from 7 centers in France were screened. We excluded 31 patients who had no indication for surgery, 32 patients with missing data (missing critical clinical data: *n* = 16, missing CT scan or MRI: *n* = 16) and 192 patients were included. The main clinical characteristics are presented in Table 1 according to cardiac surgery. Patients treated medically only without cardiac surgery show significantly higher proportions of valve prosthesis IE, lower left ventricular ejection fraction, more severe clinical forms as underlined by EuroSCORE or SOFA score, lower GCS, higher proportion of extra-cerebral localizations and septic shock.

**Table 1:** Comparison of baseline characteristics of patients

	Unmatched cohort (*n* = 192)					Propensity matched cohort (n = 92)				
	N	Cardiac surgery, *n* = 125	N	No surgery, *n* = 67	*p*	*N*	Cardiac surgery, *n* = 44	N	No surgery, *n* = 44	*p*
Age*, yr median (IQR)	125	62 (48–67)	67	65 (50–73.5)	0,10	44	65 (16.5)	44	64.5 (22)	0.56
Male*, *n* (%)	125	89 (71.2%)	67	44 (65.7%)	0,43	44	29 (65.9%)	44	28 (63.6%)	1.0
Preexisting medical conditions, *n* (%)										
Diabetes mellitus, *n* (%)	125	34 (27.2%)	67	17 (25.4%)	0,78	44	16 (36.4%)	44	10 (22.7%)	0,24
Chronic kidney disease, *n* (%)	125	48 (38.4%)	67	28 (41.8%)	0,65	44	12 (27.2%)	44	8 (18.2%)	0.52
Immunosuppression, *n* (%)	125	10 (8%)	67	11 (16.4%)	0,07	44	5 (11.4%)	44	6 (13.6%)	0,12
Valve prosthesis*, *n* (%)	125	26 (20.8%)	67	24 (35.8%)	0,02	44	13 (29.5%)	44	11 (25.0%)	0,81
Bacteriological characteristics, *n* (%)										
Positive blood cultures, *n* (%)	125	116 (92.8%)	67	58 (86.6%)	0,16	44	42 (95.5%)	44	38 (86.4%)	0,43
Positive valve culture, *n* (%)	101	35 (34.7%)	67	–	–	33	19 (51.4%)	0	–	–
Isolated bacterial specie	125		67		0.39	44		44		0.20
*Staphylococcus sp.*		67 (53.6%)		37 (55.2%)			20 (48.8%)		22 (59.5%)	0.20
- MSSA		57 (45.6%)		30 (44.8%)			18 (43.9%)		20 (54.1%)	
- MRSA		5 (4%)		4 (6%)			2 (4.9%)		2 (5.4%)	
- Others		5 (4%)		3 (4.5%)			1 (2.4%)		1 (2.7%)	
*Streptococcus sp*.		32 (26%)		10 (15%)			10 (24.4%)		5 (13.5%)	
*Enterobacterales*		10 (8%)		9 (13.4%)			4 (9.5%)		6 (15.8%)	
Others (*Enterococcus sp*., …)		14 (11.2%)		4 (6%)			4 (9.8%)		4 (10.8%)	
Health-care acquired endocarditis, *n* (%)	125	25 (20%)	67	20 (29.9%)	0.12	44	13 (29.5%)	44	14 (31.8%)	1.00
Valve damage										
Valve damage, *n* (%)*	125		67		0.74	44		44		0.50
- Mitral and aortic		24 (19%)		16 (24%)			9 (20.5%)		5 (11.4%)	
- Mitral		45 (36%)		22 (33%)			16 (36.4%)		17 (38.6%)	
- Aortic		56 (45%)		29 (43%)			19 (43.2%)		22 (50.0%)	
Medical condition at admission to ICU										
EuroSCORE mean ± SD	125	17 ± 17	67	25 ± 18	0.003	44	20 ± 20	44	21.5 ± 17	0.74
SOFA score*, median (IQR)	125	6 (4–9)	67	9 (6–13)	< 0.001	44	6 (6)	44	7.5 (7)	0.24
LVEF %, mean ± SD	125	58 ± 10	67	52 ± 13	0.001	44	58 ± 8	44	54 ± 13	0.07
Lowest Glasgow score, median (IQR)	125	14 (13–15)	67	11 (6–14)	< 0.001	44	14 (2.5)	44	11.5 (6.2)	0.03
Glasgow < 10, *n* (%)	125	21 (17%)	67	29 (43%)	< 0.001	44	17 (39%)	44	9 (21%)	0.07
Killip score, median (IQR)	114	2 (1–3)	63	2 (1–2.5)	0.32	41	1 (2)	44	2 (2)	0.78
Septic shock*, *n* (%)	125	28 (22.4%)	67	31 (46.3%)	0.001	44	16 (36.4%)	44	19 (43.2%)	0.66
Cardiogenic shock, *n* (%)	125	20 (16%)	67	14 (20.9%)	0.40	44	7 (15.9%)	44	11 (25%)	0.43
Extra neurological localizations, *n* (%)										
Extra cerebral localization	125	110 (88%)	67	66 (98.5%)	0,01	44	44 (100%)	44	44 (100%)	1.0
Primary antimicrobial therapy, *n* (%)										
Adequate, *n* (%)	117	112 (95.7%)	58	57 (98.3%)	0,38	41	38 (93%)	38	37 (97%)	0.61
Aminoglycoside, *n* (%)	125	105 (84%)	66	55 (83.3%)	0,90	44	35 (79%)	43	37 (86%)	0.60

*MSSA* methicillin-susceptible *Staphylococcus aureus*, *MRSA* methicillin-resistant *Staphylococcus aureus*, *LVEF* left ventricular ejection fraction

^*^These variables were included in the propensity score

^**^Pacemaker-associated endocarditis had multiple valve lesions including left-sided endocarditis and pacemaker endocarditis

### Neurological events

Every patient included in the study received cerebral CT scan (53%) or MRI (47%) before surgery. As shown in Table 2, ischemic stroke was the most prevalent neurological complication (74.5%) followed by hemorrhagic lesion (15.6%). Both volumes of ischemic stroke and hemorrhagic transformation were lower in patient who will have surgery.

**Table 2:** Neurological events in patients with surgical indications for infective endocarditis

Neurological events										
	Unmatched cohort					Propensity-matched cohort				
	*N*	Cardiac surgery, *n* = 125	*N*	No surgery, *n* = 67	p	*N*	Cardiac surgery, *n* = 44	*N*	No surgery, *n* = 44	*p*
MRI, *n* (%)	125	62 (49.6%)	67	28 (41.8%)	0.3	44	26 (59.1%)	44	24 (54.5%)	0.83
Ischemic stroke										
Ischemic stroke*, *n* (%)	125	96 (76.8%)	67	47 (70.1%)	0.31	44	32 (72.7%)	44	31 (70.5%)	1.00
Volume ischemic stroke, mm3, mean ± SD	96	9.5 ± 16.3	47	27.4 ± 51.2	0.03	31	9.6 ± 14.7	30	19.4 ± 30.8	0.12
Hemorrhagic transformation, *n* (%)	125	12 (9.6%)	67	8 (11.9%)	0.61	44	5 (11.4%)	44	4 (9.1%)	1.00
Volume of hemorrhagic transformation, mm3, mean ± SD	12	2.0 ± 2.0	8	10.9 ± 16.4	< 0.001	5	3.1 ± 2.5	4	10.3 ± 15.5	0.42
Hemorrhagic stroke										
Hemorrhagic stroke*, *n* (%)	125	16 (12.8%)	67	14 (20.9%)	0.14	44	5 (11.4%)	44	9 (20.5%)	0.38
Volume hemorrhagic stroke, mm3, mean ± SD	16	18.7 ± 26.4	14	10.0 ± 12.0	0.25	5	33.2 ± 42.8	9	9.1 ± 9.3	0.28
Other radiologic patterns										
Abscess, *n* (%)	125	3 (2.4%)	67	0 (0%)	0.20	44	0	44	0	1.0
Mycotic aneurysms, *n* (%)	124	16 (12.9%)	67	(2) 2.9%	0.02	44	8 (18.2%)	44	2 (4.6%)	0.09
Sub arachnoid hemorrhage, *n* (%)	125	24 (19.2%)	67	14 (20.9%)	0.78	44	6 (13.6%)	44	11 (25.0%)	0.28
Microbleeds, mean ± SD	62	5 ± 8.6	28	1.7 ± 3.1	< 0.01	20	7.9 ± 12	18	0.9 ± 1.7	0.02
Meningitis, *n* (%)	125	12 (9.6%)	67	9 (13.4%)	0.42	44	4 (9.1%)	44	7 (15.9%)	0.35
Abscess, *n* (%)	125	3 (2.4%)	67	0 (0%)	0.20	44	0	44	0	1.0
Timings										
Antimicrobial therapy to imaging diagnosis time, days, median (IQR)	125	3 (0–9)	67	1 (0–8)	0.58	44	1.5 (8)	44	6 (10)	0.14

^*^These variables were included in the propensity score

### Surgery

**Fig. 1:**
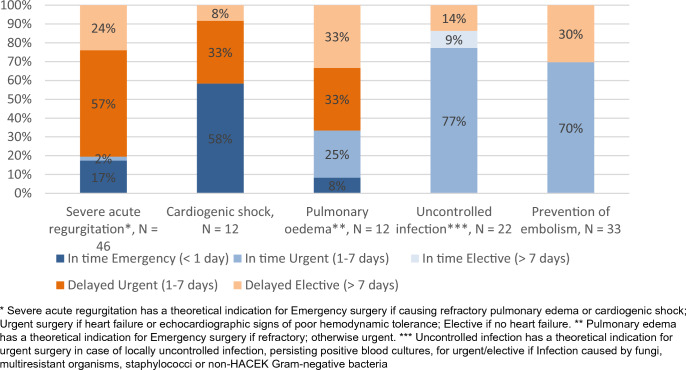
Theoretical and actual indications for cardiac surgery in infectious endocarditis. Theoretical indications are listed in x axis and delays according the recommended timing are color-coded

Among the 192 patients included, 67 (35%) patients did not receive surgery and 125 (65%) underwent cardiac surgery. The majority of indications was urgent (surgery < 7 days) (59%). The actual timing between antibiotic initiation and ICU admission was 8 days IQR [3–26] in median, and 15 days [8–35] between antibiotic initiation and surgery (Additional file 1 and Additional file 2). Indications and timings according to the surgeon’s actual decision and ESC guideline (theoretical) are shown in Table 3. The heart failure indication for surgery was more frequently observed in patients with surgery, whereas indication for embolism prevention was more frequent in patients who finally did not receive cardiac surgery. Among the 125 patients who received surgery, 62 (50%) had delayed surgery according to recommended timing. In Table 2 and Fig. 1, the comparison between actual and timing of surgery showed that patients who will have surgery present with more emergency and less urgent indications than patients who will not. The surgery was more frequently delayed (from emergency to urgent or elective) in patients with severe acute regurgitation (*n* = 37/46 (80%)), whereas patients with cardiogenic shock were less prone to be delayed (*n* = 5/12 (42%)).

**Table 3:** Operation in patients with surgical indications for infective endocarditis

	Unmatched cohort (*n* = 192)			Propensity matched cohort (*n* = 92)		
	Cardiac surgery, *N* = 125	No surgery, *N* = 67	*p*	Cardiac surgery, *N* = 46	No surgery, *N* = 46	*p*
Cardiac surgery						
Indication for cardiac surgery, *n* (%)*			< 0.001			0.79
1 Heart failure	70 (56%)	17 (25.4%)		18 (39.9%)	16 (36.4%)	
- Severe acute regurgitation	46	12		10	11	
- Cardiogenic shock	12	4		4	4	
- Pulmonary edema	12	1		4	1	
2 Uncontrolled infection	22 (17.6%)	8 (11.9%)		6 (13.6%)	7 (15.9%)	
3 Prevention of embolism	33 (26.4%)	42 (62.7%)		20 (45.5%)	21 (47.7%)	
Surgical timing, *n* (%)						
- Emergency	63 (50.4%)	17 (25.4%)	< 0.001	17 (38.6%)	16 (36.4%)	1.0
- Urgent	60 (48.0%)	48 (71.6%)		26 (59.1%)	27 61.4%)	
- Elective	2 (1.6%)	2 (2.9%)		1 (2.3%)	1 (2.3%)	
Actual surgical timing, *n* (%)						
- Emergency	20 (16.0%)	–	–	5 (11.4%)	–	–
- Urgent	74 (59.2%)	–	–	23 (52.3%)	–	–
- Elective	31 (24.8%)	–	–	16 (36.4%)	–	–
Delayed surgery, *n* (%)	62 (49.6%)	–	–	22 (50.0%)	–	

^*^This variable was included in the propensity score

### Propensity score

The distribution of the variables included in the propensity score in the unmatched and matched cohorts is presented in Additional file 3. Additional file 4 shows a large mismatch of the propensity of being operated according to whether the patients were actually operated or not.

### Analysis of the primary outcome

**Fig. 2:**
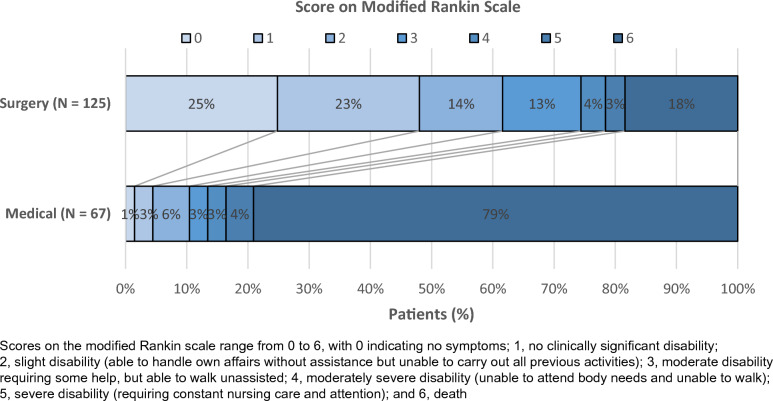
Distribution of modified Rankin scale scores at 6 months (*n* = 192 patients)

The crude proportion of favorable 6-month neurological outcome (mRS score ≤ 3) was 74.4% (*n* = 93/125) *vs* 13.4% (9/67) (*p* < 0.001) in the group of patients receiving surgery or not, respectively (Fig. 2). In the propensity score-adjusted logistic regression (*n* = 192 patients), the odds ratio (OR) for favorable 6-month neurological outcome was 13.8 (95% CI 6.2–33.7) in favor of surgery.

### Secondary outcomes

#### 6-month modified Rankin score in the matched cohort

After 1:1 propensity score matching, a total of 88 patients were still evaluable for the matched-pairs analysis. Baseline characteristics between the two groups achieved good balance (Tables 1, 3). The OR for favorable 6-month neurological outcome associated with surgery was 3.6 (95% CI 2.0–6.7).

#### 6-month modified Rankin score by IPTW analysis

The benefit of cardiac surgery in the overall population (*n* = 192 patients) was confirmed by the IPTW analysis with an OR for favorable 6-month neurological outcome associated with surgery of 20 (95% CI 11–50), *p* < 0.01).

#### 1-year mortality in the overall population (n = 192 patients)

**Fig. 3:**
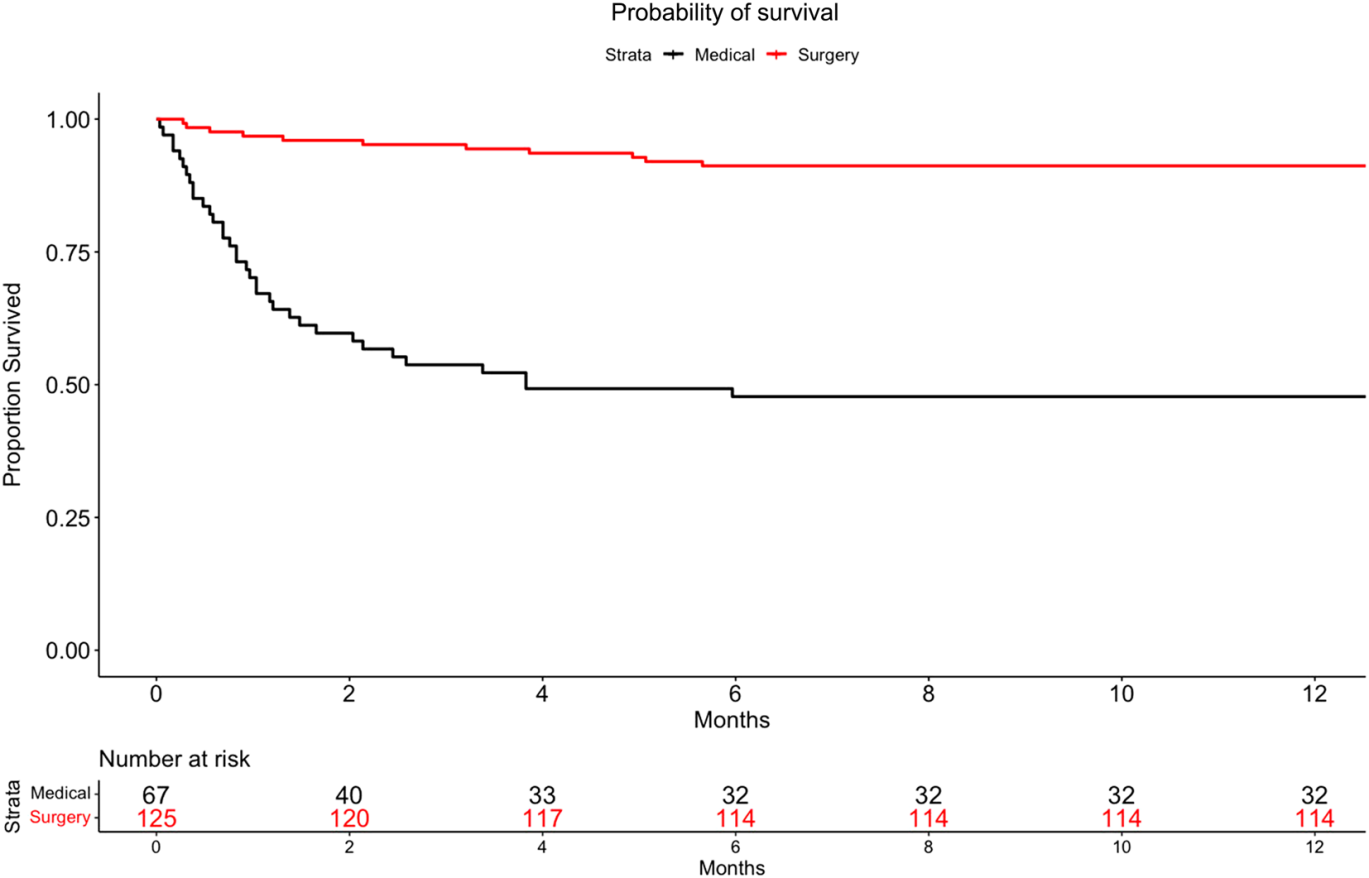
Kaplan–Meier curves of the probability of survival from inclusion to month 12

The 1-year mortality was associated with surgery in the fixed-effect adjusted Cox model (hazard ratio 0.18; 95% CI 0.11–0.27; *p* < 0.001) (Fig. 3). To assess the overall effect of time-dependent bias, Cox regression analysis was repeated with surgery as a time-dependent covariate and showed robustness.

### Subgroup analysis by timing of surgery

A subgroup analysis according to whether timing of surgery was delayed or not was performed. Among the 125 patients who received surgery, 63 were delayed (details in Additional file 5) and 62 received surgery in a timely manner. The proportion of patients with a 6-month modified Rankin scale score ≤ 3 was 84.1% in patients with delayed surgery and 77.7% in patients with timely surgery (*p* = 0.14). The 1-year mortality rate was 15.9% and 33.9% (*p* = 0.09), respectively, in these 2 groups. In a propensity score-adjusted logistic regression, delayed or timely surgery were both significantly associated with a favorable 6-month neurological outcome (mRankin score ≤ 3) when compared with no surgery (delayed surgery OR 8.2; 95% CI 2.6–15.3 and timely surgery OR 4.2; 95% CI 2.6–7.1). In the matched cohort, the 1-year mortality was, respectively, associated to delayed and timely surgery with a HR of 4.7; 95% CI 2.0–11.4 and 2.9; 95% CI 1.4–6.1.

### Subgroup analysis by baseline Glasgow Coma Scale < vs ≥ 10

A subgroup analysis according to baseline coma GCS < vs ≥ 10 was performed in the non-matched (*n* = 192) and in the matched cohort (*n* = 88). The lower risk of surgery-associated 1-year mortality observed in the entire cohort was confirmed in patients with GCS < 10 (OR 5.7; 95% CI 3.7–8.8).

### Subgroup analysis by type of stroke

The crude proportion of favorable 6-month neurological outcome (mRS score ≤ 3) was 71.3 vs 10.9% (*p* < 0.001) in ischemic strokes and 93.8 vs 7.1% (*p* < 0.001) in hemorrhagic strokes, with medical vs surgical treatment, respectively.

## Discussion

In this study including patients with simultaneous IE, cerebral events, and an indication for surgery, receiving surgery was associated with a better neurological outcome, defined by a 6-month Rankin score ≤ 3. The strength of this association was high (13.8 (95% CI 6.2–33.7)), consistent in a matched analysis and robust despite the use of several methods to account for selection bias. This association was also observed with the 1-year mortality. No difference in this association was found between patients with initial Glasgow < vs ≥ 10.

Our study has some strengths. It addresses an original issue in focusing the IE patients admitted to ICU with indications for surgery and presenting neurological events detected before surgery. This study, by including patients for whom surgery decisions were taken at the physicians’ discretion, is also representative of the real life management. By using a propensity score-adjusted analysis, confusion bias—which are very potent in this field—are limited (despite not eliminated). Moreover, survivor treatment selection bias, whereby surgery may be falsely interpreted as being associated with improved survival can be addressed by combining a fixed-effect and a time-dependent analysis [22]. Regarding patients who do not require admission to ICU, several studies assessed the association between surgery and neurological outcome using a propensity-adjusted analysis, with apparent discrepancies. In fact, if surgery was suggested to be beneficial in two studies [23, 24] surgery was not assessed as a time-dependent variable. On the contrary, medical treatment seemed to be beneficial in two other studies, one with time-dependent analysis [25]. Nevertheless, this association was not confirmed in another study with both propensity-adjusted and time-dependent analysis [22]. This analysis was not performed in previous ICU studies [9–11, 14, 26] and our study is the first study focusing on IE patients admitted to ICU, with both indications for surgery and neurological events detected before surgery using a propensity score-adjusted analysis and combining a fixed-effect and a time-dependent analysis. We hypothesized a self-fulfilling prophecy by which cerebral complications may be considered as too severe to undergo surgery which in turn may lead to a bad outcome and finally confirm the prophecy. On the contrary, this study adds to the evidence that surgery may still be beneficial in many of these patients.

Our study has some limitations. First, a selection bias is possible. In France, all IE patients with surgical indications converge to university tertiary hospitals because they are the only ones with cardiac surgery units. Despite this centralization, some patients with at least one indication for surgery might not have been referred to surgery centers after multidisciplinary discussion because of associated factors such as severe comorbidities, multi-organ failure or severe cerebral complications. A second limitation is that no systematic screening of hemorrhagic complications after surgery was performed, but we assume that they have influenced mRs at 6 months. Moreover, we did not have a specific neurological status at admission other than the GCS and specific causes of mortality or specific reason for surgery abstention were not reported or collected. The subgroup analysis of patients with hemorrhagic stroke has not the adequate power to lead to formal conclusions. The main limitation comes from the persistence of confusion bias despite the construction of a propensity score. Also, a lack of power may exists in our study, and future studies may focus on each indication of surgery to analyze their specific benefit–risk balance. Finally, the propensity score, built to adjust for the maximum of patient characteristics that may have played a role in the ethical decision (of surgery or not), cannot reflect the complexity of real ethical decisions and may have missed important information.

Most of the published studies including ICU patients with IE mixed patients with and without neurological events and thus prevent to assess the surgery-associated prognosis in the specific subset of IE patients with neurological events [9, 11, 14]. Other authors report data on patients presenting with neurological ischemic events (*n* = 556), indistinctly of their severity (requiring or not admission to ICU): 237 (43%) had surgery [26]. The two other studies in which specific data are reported for the same subgroup as ours show a close rate of surgery. Compared to 65% in our study, Sonneville et al. reported a rate of surgery of 55% (59/108) but without reporting the outcomes of the 52/59 patients with neurological events which occurred before surgery. In Rambaud et al. 108/136 (78%) were operated [10, 12]. In our study, ischemic stroke was the most prevalent neurological complication (75%) followed by hemorrhagic lesions (hemorrhagic transformations of ischemic stroke and hemorrhagic stroke) (31%). These proportions are very close to what others reported (Sonneville et al. 71%; Barsic et al. 65%) [10, 26]. The proportion of patients with GCS < 10 (26%) was also consistent with previous data (28%) [11]. In this study, coma GGS < 10 was not a factor of worse prognosis in patients receiving surgery, despite being associated with 1-year mortality[11], indicating that the deepness of coma may not be a potential contraindication to surgery.

## Conclusions

In IE patients admitted to ICU with simultaneously an indication for cardiac surgery and at least one neurological event, surgery was associated with a better 6-month neurological outcome. This association remains both in patients with Glasgow coma score < or ≥ 10.

### Supplementary Information

**Additional file 1. **Timing between antibiotic initiation and surgery.

**Additional file 2. **Timing between ICU admission and surgery.

**Additional file 3. **Distribution of variables selected in the propensity score.

**Additional file 4. **Distribution of the propensity score in the matched population according to surgical vs medical treatment.

**Additional file 5. **Indications for surgery according to different categories of delay.

## Data Availability

The datasets analyzed during the current study are available from the corresponding author on reasonable request.

## Abbreviations

CT scan: Cerebral computed tomography scan
ESC: European Society of Cardiology
GCS score: Glasgow Coma Scale score
MRI: Magnetic resonance imagery
IIA: Infectious intracranial aneurysm
IE: Infective endocarditis
ICU: Intensive care unit
IQR: Interquartile range
mRS: Modified Rankin score
OR: Odds ratio
SAH: Subarachnoid hemorrhage
SOFA: Sequential Organ Failure Assessment score
SRLF: Société de Réanimation de Langue Française
SD: Standard deviation
