# Supplementary material for: Critically ill patients with infective endocarditis, neurological complications and indication for cardiac surgery: a multicenter propensity-adjusted study
Source: Ann Intensive Care. 2024 Feb 2;14:21. doi: 10.1186/s13613-023-01221-x (PMC10837394; doi:10.1186/s13613-023-01221-x)
Supplement: Supplementary file 1 — Additional file 1. Timing between antibiotic initiation and surgery. [file 13613_2023_1221_MOESM1_ESM.docx]

Additional file 1

Figure

Timing between antibiotic initiation and surgery
