# Supplementary material for: Critically ill patients with infective endocarditis, neurological complications and indication for cardiac surgery: a multicenter propensity-adjusted study
Source: Ann Intensive Care. 2024 Feb 2;14:21. doi: 10.1186/s13613-023-01221-x (PMC10837394; doi:10.1186/s13613-023-01221-x)
Supplement: Supplementary file 2 — Additional file 2. Timing between ICU admission and surgery. [file 13613_2023_1221_MOESM2_ESM.docx]

Additional file 2

Figure

Timing between ICU admission and surgery

**SF 3**
