# Supplementary material for: Critically ill patients with infective endocarditis, neurological complications and indication for cardiac surgery: a multicenter propensity-adjusted study
Source: Ann Intensive Care. 2024 Feb 2;14:21. doi: 10.1186/s13613-023-01221-x (PMC10837394; doi:10.1186/s13613-023-01221-x)
Supplement: Supplementary file 3 — Additional file 3. Distribution of variables selected in the propensity score. [file 13613_2023_1221_MOESM3_ESM.docx]

Additional file 3

Table.

Distribution of variables selected in the propensity score

|  | Unmatched Cohort | | | Propensity Matched Cohort | | |
| --- | --- | --- | --- | --- | --- | --- |
|  | Cardiac Surgery, N = 125 | No surgery, N = 67 | p | Cardiac surgery,  N = 44 | No surgery,  N = 44 | p |
| Age, yr, median (IQR) | 62 (48-67) | 65 (50-73) | 0.1 | 65 (16.5) | 64.5 (22) | 0.16 |
| Male, n (%) | 89 (71.2%) | 44 (65.7%) | 0.43 | 29 (65.9%) | 28 (63.6%) | 1.0 |
| Valve prosthesis, n (%) | 26 (20.8%) | 24 (35.8%) | 0.02 | 13 (29.5%) | 11 (25.0%) | 0,81 |
| Valve damage, n (%)   - Mitral and aortic - Mitral - Aortic | 24 (19.2%)  44 (35.2%)  56 (44.8%) | 16 (23.9%)  22 (32.8%)  29 (43.3%) | 0.76 | 9 (20.5%)  16 (36.4%)  19 (43.2%) | 5 (11.4%)  17 (38.6%)  22 (50.0%) | 0.50 |
| SOFA score, median (IQR) | 6 (4-9) | 9 (6-13) | <0.001 | 6 (6) | 7 .5 (7) | 0.92 |
| Septic shock | 28 (22.4%) | 31 (46.3%) | 0.001 | 16 (36.4%) | 19 (43.2%) | 0.66 |
| Ischemic stroke*, n | 96 (76.8%) | 47 (70.1%) | 0.31 | 32 (72.7%) | 31 (70.5%) | 1.00 |
| Haemorrhagic stroke*, n (%) | 16 (12.8%) | 14 (20.9%) | 0.14 | 5 (11.4%) | 9 (20.5%) | 0.38 |
| Indication for cardiac surgery, n (%) <0.001 0.79 | | | | | | |
| 1. Heart failure  - Severe acute regurgitation - Cardiogenic shock - Pulmonary oedema | 70 (56%)  46  12  12 | 17 (25.4%)  12  4  1 |  | 18 (39.9%)  10  4  4 | 16 (36.4%)  11  4  1 |  |
| 1. Uncontrolled infection | 22 (17.6%) | 8 (11.9%) |  | 6 (13.6%) | 7 (15.9%) |  |
| 1. Prevention of embolism | 33 (26.4%) | 42 (62.7%) |  | 20 (45.5%) | 21 (47.7%) |  |
