# Supplementary material for: Critically ill patients with infective endocarditis, neurological complications and indication for cardiac surgery: a multicenter propensity-adjusted study
Source: Ann Intensive Care. 2024 Feb 2;14:21. doi: 10.1186/s13613-023-01221-x (PMC10837394; doi:10.1186/s13613-023-01221-x)

Additional file 4

Figure

Distribution of the propensity score in the matched population according to surgical vs medical treatment


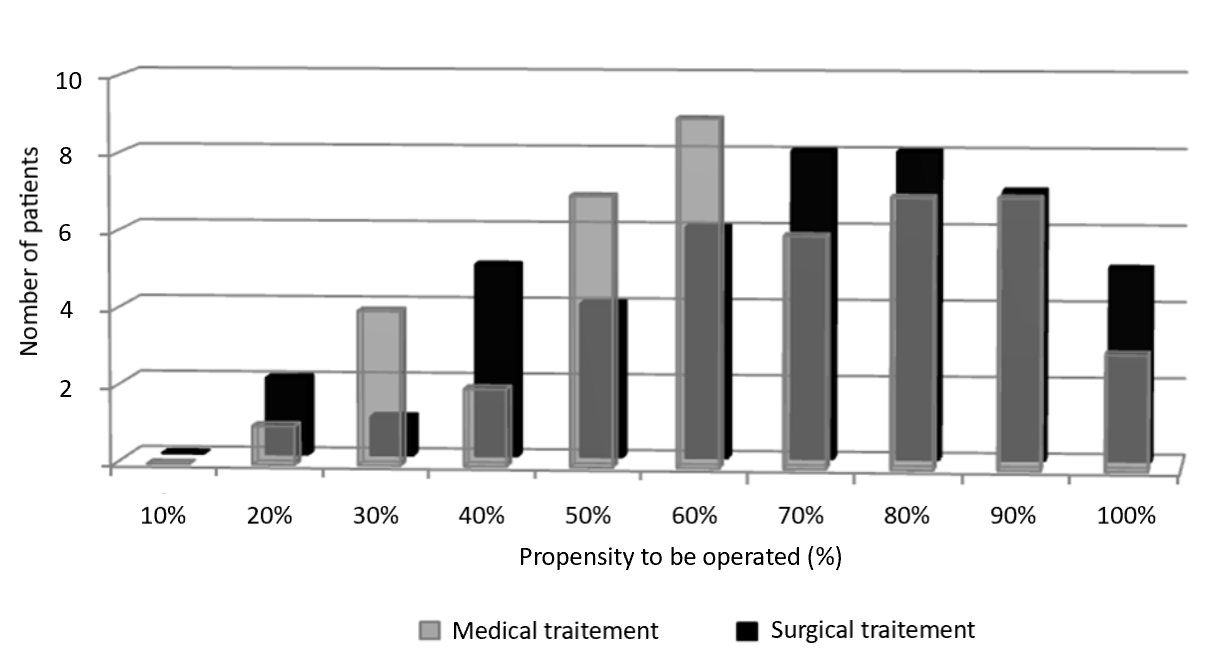

Supplement: Supplementary file 4 — Additional file 4. Distribution of the propensity score in the matched population according to surgical vs medical treatment. [file 13613_2023_1221_MOESM4_ESM.docx]
