# Supplementary material for: Critically ill patients with infective endocarditis, neurological complications and indication for cardiac surgery: a multicenter propensity-adjusted study
Source: Ann Intensive Care. 2024 Feb 2;14:21. doi: 10.1186/s13613-023-01221-x (PMC10837394; doi:10.1186/s13613-023-01221-x)
Supplement: Supplementary file 5 — Additional file 5. Indications for surgery according to different categories of delay. [file 13613_2023_1221_MOESM5_ESM.docx]

Additional file 5.

Table . Indications for surgery according to different categories of delay.

|  | Emergency 🡪 urgent  n=34 | | | Emergency 🡪 elective  n=13 | | | Urgent 🡪 elective  n=16 | | |
| --- | --- | --- | --- | --- | --- | --- | --- | --- | --- |
|  | N(%) | Modified Rankin score > 3, 6 months | Mortality, 1 year | N(%) | Modified Rankin score, 6 months | Mortality, 1 year | N(%) | Modified Rankin score, 6 months | Mortality, 1 year |
| Heart failure, n (%) or mean (SD)   - Severe acute regurgitation - Cardiogenic shock - Pulmonary oedema | 34 (100)  26 (76)  4 (12)  4 (12) | 9 (27)  5 (15)  2 (6)  2 (6) | 9 (27)  4 (12)  3 (9)  2 (6) | 13 (100)  11 (84)  1 (8)  1 (8) | 1 (8)  1 (8)  0 (0)  0 (0) | 1 (8)  1 (8)  0 (0)  0 (0) | 3^a^(19)  0 (0)  0 (0)  3 (19) | 0 (0)  0 (0)  0 (0)  0 (0) | 0 (0)  0 (0)  0 (0)  0 (0) |
| Uncontrolled infection | 0 (0) | 0 (0) | 0 (0) | 0 (0) | 0 (0) | 0 (0) | 3 (19) | 0 (0) | 0 (0) |
| Embolism prevention | 0 (0) | 0 (0) | 0 (0) | 0 (0) | 0 (0) | 0 (0) | 10 (81) | 2 (13) | 2 (13) |

^a^ Some heart failure cases are indications for urgent rather than emergency indications (e.g. urgent indication in case of aortic or mitral native valve endocarditis or prosthetic valve endocarditis with severe regurgitation or obstruction causing symptoms of HF or echocardiographic signs of poor haemodynamic tolerance. Emergency indication in case of refractory pulmonary oedema or cardiogenic shock associated).
